# Conversion of fatty aldehydes into alk (a/e)nes by *in vitro* reconstituted cyanobacterial aldehyde-deformylating oxygenase with the cognate electron transfer system

**DOI:** 10.1186/1754-6834-6-86

**Published:** 2013-06-08

**Authors:** Jingjing Zhang, Xuefeng Lu, Jian-Jun Li

**Affiliations:** 1 Key Laboratory of Biofuels, Shandong Provincial Key Laboratory of Energy Genetics, Qingdao Institute of Bioenergy and Bioprocess Technology, Chinese Academy of Sciences, No. 189 Songling Road, Qingdao, 266101, China; 2 University of Chinese Academy of Sciences, Beijing, 100049, China

**Keywords:** Biofuels, Fatty alk(a/e)ne, *Synechococcus elongatus* PCC7942, Aldehyde-deformylating oxygenase, Ferredoxin, Ferredoxin-NADP^+^ reductase, The cognate reducing system

## Abstract

**Background:**

Biosynthesis of fatty alk(a/e)ne in cyanobacteria has been considered as a potential basis for the sunlight-driven and carbon-neutral bioprocess producing advanced solar biofuels. Aldehyde-deformylating oxygenase (ADO) is a key enzyme involved in that pathway. The heterologous or chemical reducing systems were generally used in *in vitro* ADO activity assay. The cognate electron transfer system from cyanobacteria to support ADO activity is still unknown.

**Results:**

We identified the potential endogenous reducing system including ferredoxin (Fd) and ferredoxin-NADP^+^ reductase (FNR) to support ADO activity in *Synechococcus elongatus* PCC7942. ADO (Synpcc7942_1593), FNR (SynPcc7942_0978), and Fd (SynPcc7942_1499) from PCC7942 were cloned, overexpressed, purified, and characterized. ADO activity was successfully supported with the endogenous electron transfer system, which worked more effectively than the heterologous and chemical ones. The results of the hybrid Fd/FNR reducing systems demonstrated that ADO was selective against Fd. And it was observed that the cognate reducing system produced less H_2_O_2_ than the heterologous one by 33% during ADO-catalyzed reactions. Importantly, *k*_*cat*_ value of ADO 1593 using the homologous Fd/FNR electron transfer system is 3.7-fold higher than the chemical one.

**Conclusions:**

The cognate electron transfer system from cyanobacteria to support ADO activity was identified and characterized. For the first time, ADO was functionally *in vitro* reconstituted with the endogenous reducing system from cyanobacteria, which supported greater activity than the surrogate and chemical ones, and produced less H_2_O_2_ than the heterologous one. The identified Fd/FNR electron transfer system will be potentially useful for improving ADO activity and further enhancing the biosynthetic efficiency of hydrocarbon biofuels in cyanobacteria.

## Background

It is imperative to develop renewable biofuels due to concerns about climate change, the diminishing supplies of fossil fuels, and energy security [1-6]. With regard to the sustainability of biomass resources, cellulosic ethanol and microalgal biodiesel have been becoming more and more attractive [1,6,7]. Taking into account fuel performance, ideal fuels should have very similar energy content, storage and transportation properties, and combustion properties to current transportation fuels allowing them to be used in the existing gasoline, diesel, and jet engines [3]. Fatty-acid-derived biofuels fulfil these criteria, among which fatty-acid-derived alk(a/e)nes could be the ideal replacement for fossil-based fuel due to the fact that fatty alk(a/e)nes are the main components of conventional fuels such as gasoline, diesel, and jet fuel [4]. Therefore, it is of great importance to investigate biosynthesis of fatty alk(a/e)nes.

Fatty alk(a/e)nes are mainly produced by plants, insects, birds, green algae, and cyanobacteria [8]. Cyanobacteria are the advantageous organisms over others for industrial applications as they incorporate the favourable characteristics of prokaryotes and plants, which can efficiently convert solar energy and carbon dioxide into biofuels in one biological system [2,9-11]. Furthermore, the genetic engineering platform for cyanobacteria has been well established [2,9-11].

**Figure 1:**
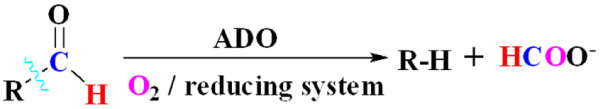
**ADO-catalysed reaction [**[8]**,**[12]**-**[16]**].** Oxygen and the auxiliary reducing system (biological or chemical) providing four electrons are needed for ADO activity, one O-atom is incorporated into formate, and the aldehyde hydrogen is retained in formate.

Fatty alk(a/e)nes produced by organisms are typified by an odd number of carbons. More and more evidence has indicated that a two-step pathway for fatty alk(a/e)ne biosynthesis exists, including: (1) reduction of fatty acyl-ACP or –CoA into corresponding aldehyde by acyl-ACP reductase; (2) conversion of fatty aldehyde into alk(a/e)ne by aldehyde decarbonylase [12]. Since it has been observed that the C1-derived coproduct of the second step is carbon monoxide, the enzyme involved in that reaction has been tentatively designated as aldehyde decarbonylase [12]. Recently, Schirmer *et al.* identified two genes involved in alk(a/e)ne biosynthesis in cyanobacteria [8]. Since identification of the biosynthetic pathway of alk(a/e)ne in cyanobacteria, aldehyde decarbonylase has attracted particular interest in industry and academia for biofuel production due to the difficult and unusual reaction it catalyses. It has been proved that: (1) the C1-derived coproduct is formate instead of widely supposed carbon monoxide (Figure 1) [12]; (2) the aldehyde hydrogen is retained in formate and the hydrogen of the nascent methyl group originates at least in part from solvent (H_2_O) [12]; (3) oxygen is absolutely required for this apparently hydrolytic reaction, and one O-atom is incorporated into formate, so it has been proposed that widely accepted aldehyde decarbonylase should be redesignated as aldehyde-deformylating oxygenase (ADO) by Li *et al.* (Figure 1) [13,14]; (4) the auxiliary reducing system (biological or chemical) providing four electrons is absolutely needed for ADO activity (Figure 1) [8,14-16]; (5) based on the crystal structure of ADO from *Prochlorococcus marinus* MIT9313, ADO belongs to the ferritin-like non-heme dimetal-carboxylate enzyme family [8,17]; (6) Andre *et al.* reported that ADO is reversibly inhibited by H_2_O_2_ originating from poor coupling of reductant consumption with alkane formation, and the inhibition can be relieved by supplementing catalase (The paper was published when the manuscript was under review.) [18].

As mentioned above, the biological or chemical reducing system is required for catalytic activity of ADO [8,14-16]. The commonly used chemical reducing system is phenazine methosulfate (PMS) or 1-methyoxy-5-methylphenazinium methylsulfate (MeOPMS) and NADH [14-16]. The widely used biological reducing system is surrogate ferredoxin (Fd) and ferredoxin-NADP^+^ reductase (FNR) from spinach, and NADPH [8,14-16]. It was observed that the chemical reducing system worked better than the biological one [14,15]. Fd from *Zea mays* and FNR from *Anabaena* sp. PCC7120 have also been used to support ADO from *Prochlorococcus marinus* MIT9313 [18].

In addition to supporting ADO ( Fd and FNR from spinach, Fd from *Zea mays* and FNR from *Anabaena* sp. PCC7120), the surrogate electron transfer systems also supported reactions catalysed by stearoyl acyl carrier protein Δ^9^ desaturase (Fd and FNR from spinach) [19], *p*-aminobenzoate *N*-oxygenase (AurF) (Fd and FNR from *Anabaena* sp. PCC7119) [20], and cytochrome P450 (P450) [21,22], etc.. P450 showed enormous diversity in the redox partner systems [23]. The homologous Fd/FNR systems generally supported greater P450 activity than the heterologous ones, implying that the interaction of Fd with FNR and P450 is very important for efficient electron transfer [24-26].

**Figure 2:**
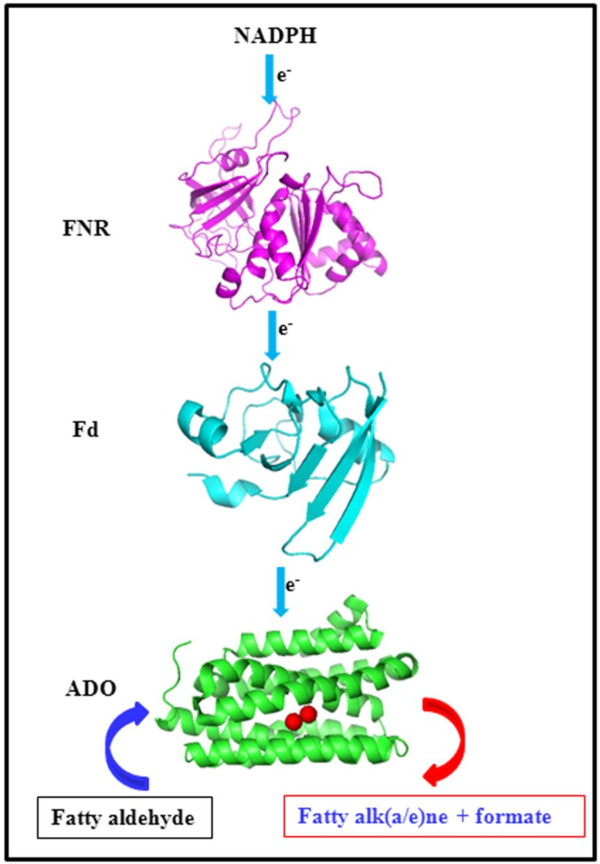
**Representative electron flow in conversion of fatty aldehyde into alk(a/e)ne with the reconstituted ADO/Fd/FNR system.** Reducing equivalents from NADPH are transferred from FNR to Fd and then to ADO. The crystal structures of related ADO (PDB ID: 2OC5, 60% sequence identity to ADO from PCC7942) from *Prochlorococcus marinus* MIT9313, Fd (PDB ID: 1QT9, 82% sequence identity to Fd from PCC7942) and FNR (PDB ID: 1QUE, 67% sequence identity to FNR from PCC7942) from *Anabaena sp.* PCC7119 were used to demonstrate electron transfer in the reconstituted ADO/Fd/FNR system.

Considering that the surrogate electron transfer system might not be well matched with cyanobacterial ADO, it is very necessary to search for the cognate one to support ADO. In this paper, ADO and the endogenous reducing system including Fd and FNR from *Synechococcus elongatus* PCC7942 were cloned, overexpressed, purified, and characterized. We reported the first cognate reducing system from cyanobacteria to support ADO activity (Figure 2). ADO was successfully *in vitro* reconstituted with the endogenous Fd/FNR system, which performed more effectively than the surrogate Fd/FNR one and the chemical reducing system.

## Results and discussion

### Searching for the endogenous electron transfer system to support ADO activity in the genome sequence of *Synechococcus elongatus* PCC7942

According to the report by Schirmer *et al*., *in vitro* enzymatic activity of ADO was only observed in the presence of Fd, FNR, and NADPH, while omitting any one of these cofactors completely abolished ADO activity [8]. However, the endogenous electron transfer system from cyanobacteria to support ADO-catalysed reaction is still unknown. In order to *in vitro* reconstitute ADO activity, it is essential to search for such a cognate electron transfer system to be well matched with ADO in cyanobacteria.

Two FNR isoforms were isolated in *Synechocystis sp.* PCC6803, the smaller one (FNR_S_) similar to the one found in plant plastids and the larger one (FNR_L_) associated with the phycobilisome, both of which derive from the single FNR gene (*petH*), and the smaller one is produced from the second translation initiation site within the *petH* ORF [27,28]. Two FNR isoforms were also detected in *Anabaena sp.* PCC7120 and *Synechococcus sp.* PCC7002 [28]. However, the smaller isoform is not present in cyanobacteria lacking a second methionine within the *petH* ORF such as *Synechococcus elongatus* and *Thermosynechococcus elongatus*[28], so only one FNR isoform is present in PCC7942. According to the genome sequence of PCC7942 (http://genome.microbedb.jp/cyanobase/SYNPCC7942), FNR is encoded by the *SynPcc7942_0978* (*petH*) gene, and would be involved in electron transfer between FNR and its protein partners in PCC7942.

There are at least seven Fd and Fd-like proteins in the genome sequence of PCC7942 (http://genome.microbedb.jp/cyanobase/SYNPCC7942), among which Ferredoxin I (SynPcc7942_1499) encoded by the *petF* gene was found to be indispensable for PCC7942 [29]. In addition, in PCC6803 the corresponding *petF* gene (ssl0020) coding for the most abundant ferredoxin product was also proved to be critical to cell growth, and the expression level of the *petF*-like genes such as sll1382, slr0150 and slr1828 is very weak compared with that of *petF*[30]. So, we think that Fd encoded by the *petF* gene is very important for a lot of redox processes in cyanobacteria, such as mediating electron transfer from iron–sulphur centres of photosystem I to FNR which then reduces NADP^+^ for CO_2_ fixation, cyclic photophosphorylation, nitrogen assimilation, sulphite reduction or fatty acid metabolism, etc.. Based on these considerations, Fd (SynPcc7942_1499) encoded by the *petF* gene was chosen for mediating electron transfer between FNR and ADO.

Therefore, FNR (SynPcc7942_0978) and Ferredoxin I (SynPcc7942_1499) will be investigated to support ADO from PCC7942 in the paper.

### Cloning, overexpression, purification, and characterization of FNR from *Synechococcus elongatus* PCC7942

The gene encoding FNR (SynPcc7942_0978) was amplified from genomic DNA of PCC7942 by PCR, cloned into the vector pET-28a(+) at the restriction sites of *Nde*I and *Xho*I, and overexpressed in *E. coli* BL21(DE3) under IPTG induction. Overexpressed FNR with the N-terminal His-tag was purified to homogeneity on Nickel column (Additional file 1: Figure S1). The predicted molecular weight of FNR is 44.4 kDa, corresponding very well to SDS-PAGE (Additional file 1: Figure S1). Protein yield was about 12 mg/L.

**Figure 3:**
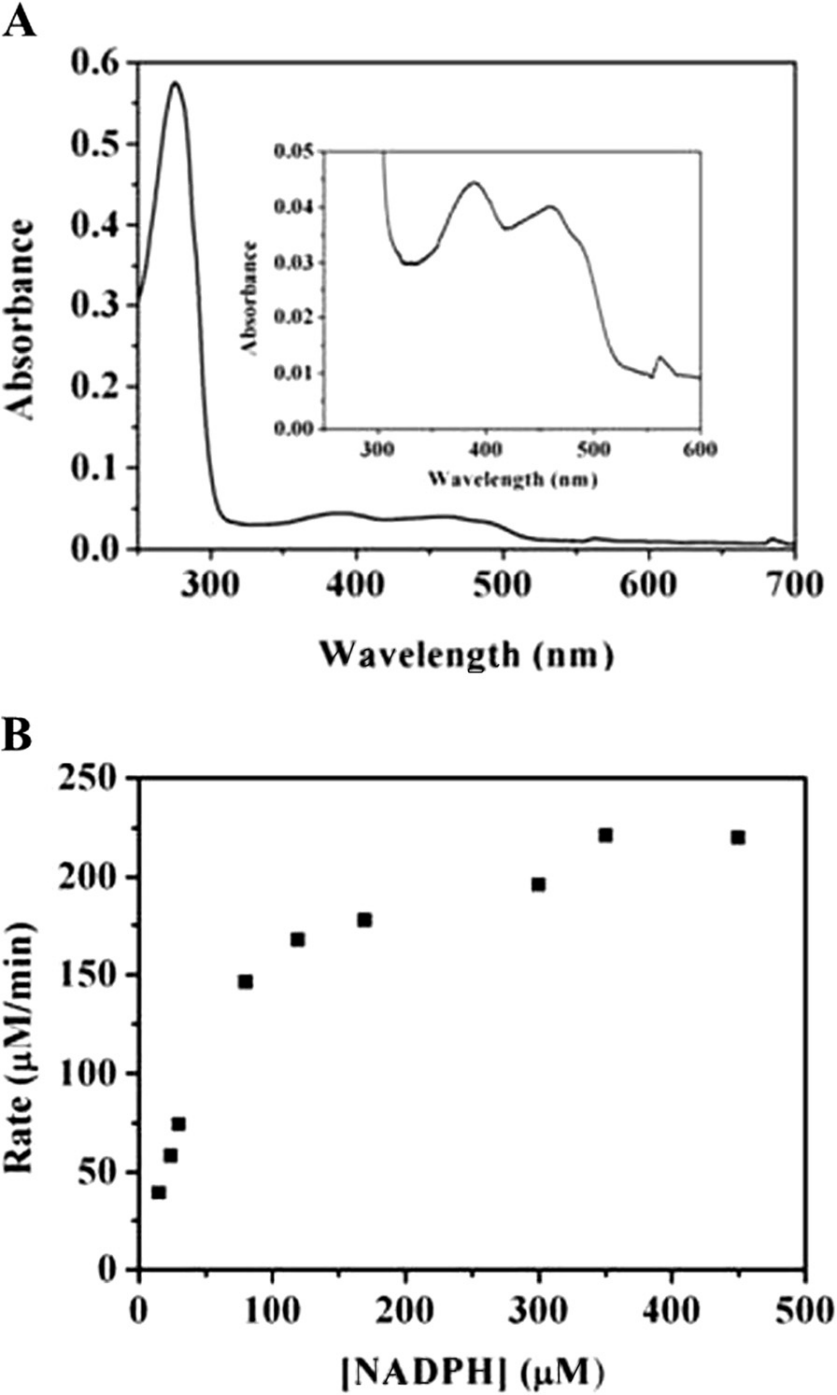
**Characterization of FNR from *Synechococcus elongatus *PCC7942.** (**A**) UV–vis absorption spectrum of FNR. (**B**) Ferricyanide reductase activity of FNR.

FNRs, usually obtained in the oxidized state, contain the noncovalently bound FAD cofactor. The released FAD from the FNR_L_-phycocyanin complex (the larger FNR isoform, 63% sequence identity to FNR from PCC7942) of PCC6803 was recovered and quantified [27]. What’s more, based on the crystal structure of FNR (PDB ID:2B5O, 61% sequence identity to FNR from PCC7942) from *Synechocystis sp.* PCC7002 with FAD bound, the residues involved in FAD binding were identified, including Arg179, Leu180, Tyr181, Ser182, Cys200, Arg202, Leu204, Tyr206, Gln207, Val218, Cys219, Ser220, Thr260, Tyr402, which are completely conserved in FNRs from PCC 7942 and PCC6803 (Additional file 2). Therefore FNR from PCC7942 should have the characteristic FAD absorption spectrum. The UV/vis spectrum of FNR clearly showed two peaks at 385 and 455 nm, and a shoulder at 480 nm, demonstrating that FNR is certainly a flavoprotein (Figure 3A) [31,32].

FNR was assayed through ferricyanide reductase activity. This assay (also called diaphorase activity) was used to determine kinetic parameters of FNR in the presence of the electron acceptor potassium ferricyanide. FNR from PCC7942 was active against potassium ferricyanide, and the initial rates were plotted against NADPH concentration and fitted according to the Michaelis-Menten equation (Figure 3B). Kinetic parameters of FNRs from different cyanobacteria were listed in Table 1. Compared with two FNR isoforms from PCC6803, FNR from PCC7942 showed higher *K*_*m(NADPH)*_ and *k*_*cat*_ values, but slightly lower catalytic efficiency [27]. In comparison with FNR from *Anabaena sp.* PCC7119, FNR from PCC7942 exhibited higher *K*_*m(NADPH)*_ and similar *k*_*cat*_ values, but much lower catalytic efficiency [33]. These results indicated that FNRs from the evolutionarily diverse classes of cyanobacteria had different binding affinity for NADPH and catalytic efficiency towards the ferricyanide reduction. In addition, it was observed that the specific activity of FNR using NADPH (375 μM) was 23-fold higher than NADH (2 mM), demonstrating that, like other FNRs [31,34], FNR from PCC7942 also prefers NADPH over NADH.

**Table 1:** Comparison of kinetic parameters of FNRs from different cyanobacteria

**FNR**	**Diaphorase activity**			**Cytochrome *c *reductase activity**			**Reference**
	***K***_***m(NADPH)***_	***k***_***cat***_	***k***_***cat***_**/*K***_***m***_	***K***_***m(Fd)***_	***k***_***cat***_	***k***_***cat***_**/*K***_***m***_	
	**(μM)**	**(s**^**-1**^**)**	**(μM**^**-1**^ **s**^**-1**^**)**	**(μM)**	**(s**^**-1**^**)**	**(μM**^**-1**^ **s**^**-1**^**)**	
PCC7942	92.5 ± 7.6	227 ± 7	2.5 ± 0.3	15.9 ± 1.2	85.6 ± 3	5.4 ± 0.5	this study
FNR_L_-PC from PCC6803	40 ± 3	124 ± 3	3.1 ± 0.3	47 ± 6	144 ± 12	3.1 ± 0.7	[27]
FNR_s_ from PCC6803	55 ± 5	174 ± 5	3.2 ± 0.4	28 ± 2	154 ± 6	5.5 ± 0.6	[27]
PCC7119	23.0 ± 1.2	225 ± 3	9.8 ± 0.2	11.0 ± 2.0	200 ± 10	18.2 ± 1.0	[33]

### Cloning, overexpression, purification, and characterization of Fd from *Synechococcus elongatus* PCC7942

The *petF* gene encoding Fd (SynPcc7942_1499) was amplified from genomic DNA of PCC7942 by PCR, cloned into the vector pET-28a(+) at the restriction sites of *Nde*I and *Xho*I, and overexpressed in *E. coli* BL21(DE3) in M9 medium under IPTG induction at 30°C [35]. Overexpressed Fd was purified on Nickel column. The predicted molecular weight of Fd with the N-terminal His-tag is 12.8 kDa, which is much smaller than the estimated one (above 20 kDa) by SDS-PAGE (Additional file 1: Figure S2). This is probably due to the acidic character of Fd (predicted pI 3.8), which prevents proper binding of SDS [36]. Fd expression was also confirmed by Western blot (data not shown). Protein yield was about 5 mg/L.

**Figure 4:**
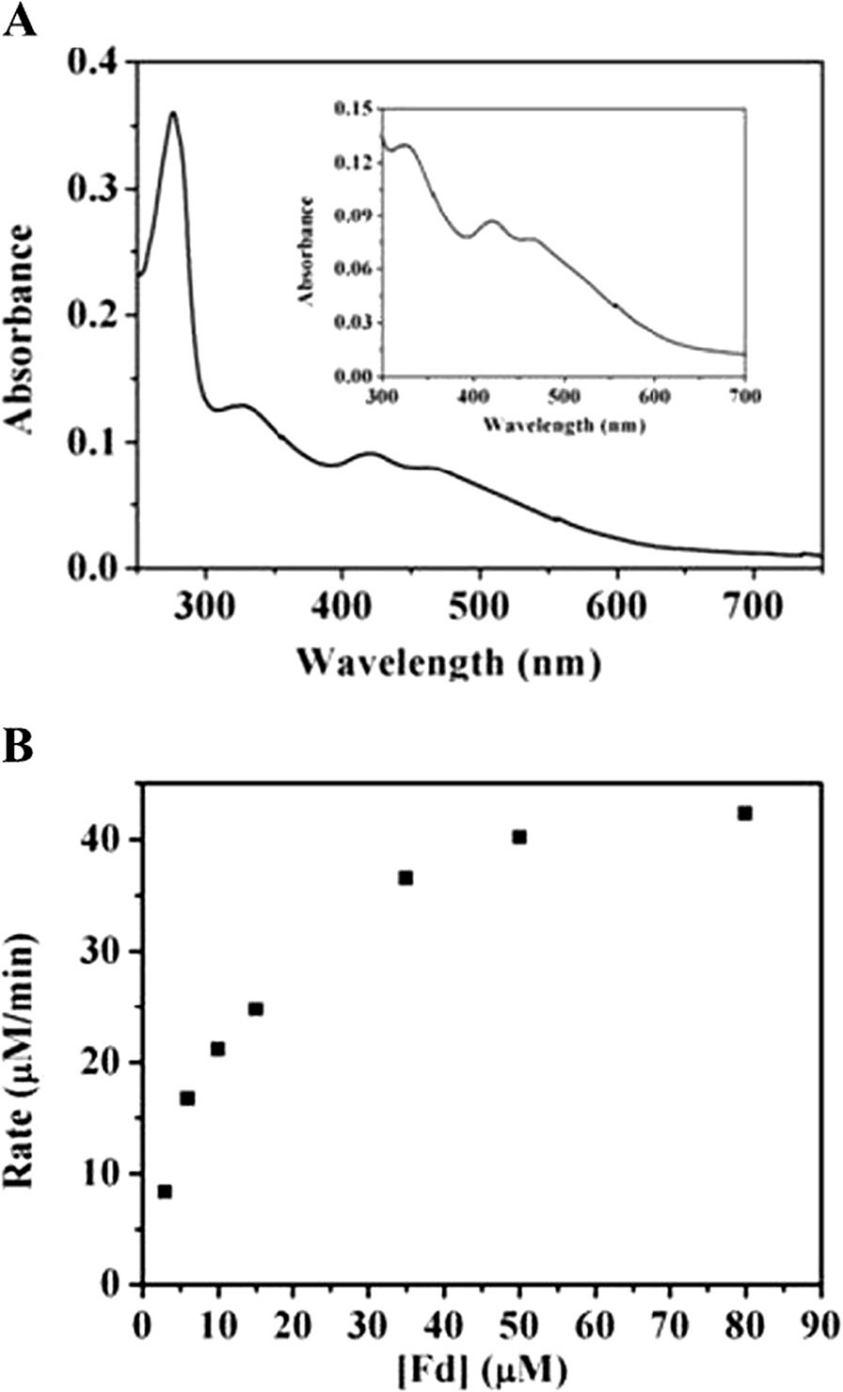
**Characterization of Fd from *Synechococcus elongatus *PCC7942.** (**A**) UV–vis absorption spectrum of Fd. (**B**) Fd-mediated cytochrome *c* reductase activity of FNR.

Based on analytical ultracentrifugation analysis, the estimated molecular weight of Fd is about 11.8 kDa after the N-terminal His-tag was removed with thrombin (Four amino acids GSHM were left at the N-terminal.), demonstrating that Fd is a monomer. MALDI-TOF-MS showed the molecular weight is 10932.79 Da after tag removal, corresponding to the predicted molecular weight 10933.02 Da. In addition, the UV/vis spectrum of Fd clearly showed three peaks characteristic of the presence of the [2Fe-2S] cluster at 331, 423, and 463 nm (Figure 4A) [36,37].

The function of Fd was investigated through Fd-mediated cytochrome *c* reductase activity of FNR. This assay was used to determine the affinity for Fd and *k*_*cat*_ value of FNR in the presence of its cognate electron acceptor Fd, and to see if the cognate FNR/Fd system from PCC7942 can be functionally coupled for efficient electron transfer.To get more information about the Fd reduction, the initial rates were obtained by varying Fd concentration under saturating concentrations of NADPH and cytochrome *c*. The initial rates of the cytochrome *c* reduction were plotted against Fd concentration and fitted according to the Michaelis-Menten equation (Figure 4B). *K*_*m(Fd)*_, *k*_*cat*_ and *k*_*cat*/_*K*_*m*_ values of FNR against the Fd-mediated cytochrome *c* reduction were 15.9 μM, 85.6 s^-1^, and 5.4 μM^-1^ s^-1^ respectively, indicating that the cognate FNR/Fd electron transfer system from PCC7942 can be efficiently and functionally coupled for the cytochrome *c* reduction. In contrast with two FNR isoforms from PCC6803, FNR from PCC7942 showed higher affinity for Fd, lower *k*_*cat*_ value, and similar catalytic efficiency to FNR_s_, but higher catalytic efficiency than FNR_L_-PC (Table 1) [27]. Compared with FNR from PCC7119, FNR from PCC7942 exhibited similar affinity for Fd, much lower *k*_*cat*_ value and catalytic efficiency (Table 1) [33]. These results also demonstrated that FNRs from different cyanobacteria exhibited different kinetic behaviour against the Fd-mediated reduction of cytochrome *c*.

### Cloning, overexpression, purification, and characterization of ADO from *Synechococcus elongatus* PCC7942

**Figure 5:**
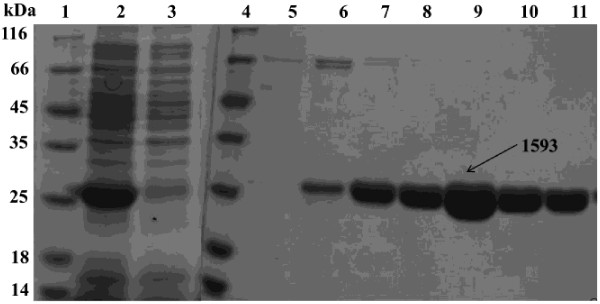
**SDS-PAGE analysis of ADO 1593.** Lane 1 and 4, low protein molecular weight marker; Lane 2, crude supernatant for over-expressed AD 1593; Lane 3, flow-through when loaded; Lane 5 and 6, eluents of buffer A + 60 mM imidazole; Lanes 7–11, eluents of buffer A + 80 mM imidazole.

The codon-optimized gene encoding ADO (Synpcc7942_1593) from PCC7942 was cloned into the vector pET-28a(+) at the restriction sites of *Nde*I and *Xho*I, and successfully overexpressed in *E. coli* BL21(DE3) under IPTG induction. Overexpressed ADO 1593 was purified to homogeneity by Nickel-chelating affinity chromatography. The target protein was eluted with buffers containing 60 and 80 mM imidazole (Figure 5). The predicted molecular weight of ADO 1593 is 26.4 kDa, corresponding well to SDS-PAGE (Figure 5). The collected fractions containing the target protein were combined and treated as reported [15,16]. Protein yield was about 15 mg/L.

Long and short-chain aldehydes such as *n*-octadecanal and *n*-heptanal were generally used as the substrates for ADO assay [8,12-16]. However, we found that *n*-hexadecanal is a better substrate than *n*-octadecanal in terms of solubility and activity (unpublished result). According to the recent paper by Andre *et al.*, actually ADO doesn’t show any strong chain-length specificity against its substrates (C8 to C18) [18]. So both *n*-hexadecanal and *n*-heptanal were tested as the substrates for ADO 1593 in the paper. Just like other ADOs, ADO 1593 was also active against these two substrates in the presence of the chemical reducing system (PMS and NADH) (data not shown).

### Comparing the effects of different reducing systems on ADO activity

#### Using *n*-hexadecanal as the substrate

We were inspired by the Fd-mediated the cytochrome *c* reduction with FNR (Figure 4B), and ADO 1593 was *in vitro* reconstituted with Fd and FNR from PCC7942. Interestingly, *n*-hexadecanal was successfully converted into *n*-pentadecane by *in vitro* reconstituted ADO 1593 with the cognate Fd/FNR system. The performance of the cognate electron transfer system in conversion of *n*-hexadecanal was then compared with the surrogate Fd/FNR system from spinach and the chemical one (PMS/NADH). The hybrid systems consisting of Fd_PCC7942_/FNR_spinach_ and Fd_spinach_/FNR_PCC7942_ were also investigated (Table 2). Assays were carried out with FNR/Fd/ADO 1593 ratios of 1:5.7:11.1 as a compromise for minimizing the yield variations with changes in the ratios [24,25].

**Table 2:** Comparison of the effects of different reducing systems on ADO activity

**Reducing system**		**Yield of *n*-pentadecane (μM)**
Chemical	PMS + NADH	8.6 ± 0.1
Biological	Fd_spinach_ + FNR_spinach_ + NADPH	15.7 ± 0.5
	Fd_spinach_ + FNR_PCC7942_ + NADPH	13.9 ± 0.4
	Fd_PCC7942_ + FNR_spinach_ + NADPH	17.6 ± 0.3
	Fd_PCC7942_ + FNR_PCC7942_ + NADPH	20.0 ± 0.8

The reaction mixtures contained 20 μM ADO 1593, 80 μM ferrous ammonium sulfate, 150 μM *n*-hexadecanal, 75 μM PMS and 750 μM NADH, or 10.26 μM Fd from spinach or PCC7942 and 1.8 μM FNR from spinach or PCC7942 and 2 mM NADPH, in 500 μL of 100 mM HEPES (pH 7.2) containing 0.1 M KCl and 10% glycerol. The reactions were incubated at room temperature for 1 hr at 200 rpm, and the products were extracted with 500 μL ethyl acetate for GC-MS analysis.

The experimental results were shown in Table 2, and at least three conclusions were drawn as listed here: (1) The biological reducing system including the cognate and surrogate ones is superior to the chemical one, which is contradictory to the published results, especially for the heterologous Fd/FNR system from spinach. The different conclusion might arise from the fact that more spinach FNR up to 1.9 U/ml was used in our assay, whereas much less FNR (0.1 U/ml or 0.04 U/ml, equivalent to less than ours by 19 or 47.5-fold) was used in the literature [14,15]. Whereas Andre *et al.* found that no more products formed when more FNR or Fd was added to an exhausted *in vitro* ADO reaction, more products were produced only when more ADO was added [18]. Considering that ADO had been completely inactivated by *in situ*-produced H_2_O_2_, this is understandable. However, the scenario is different in our case, since more FNR and Fd were used when ADO-catalyzed reactions were initiated. (2) The cognate biological reducing system is more effective than the surrogate one. *n*-Pentadecane yield using the cognate Fd/FNR system from PCC7942 is 26.6% higher than the surrogate one from spinach, implying that the interaction of Fd with FNR and ADO is very important in supporting ADO activity and mediating efficient electron transfer between Fd and its redox partners FNR and ADO. Similar results have been reported for some P450s. For example, the cognate Fd/FNR system from *Sphingomonas* sp. strain AO1 is more effective in supporting P450 than the heterologous one from spinach [26]; Ewen *et al.* recently reported that the protein–protein recognition in the mitochondrial cytochrome P450 system and modulation of electron transfer between Adx (adrenodoxin) and its redox partners AdR (adrenodoxin reductase) and cytochrome P450 are essential for mammalian cytochrome P450 [38]; When the P450 enzymes from *N. aromaticivorans* were reconstituted with the cognate ArR (Fd reductase) and Arx (a [2Fe-2S] Fd) , the steady-state turnover rates increased by 50% to 400% over those observed previously with the surrogate PdR (putidaredoxin reductase) [24,25]. (3) The hybrid experiments of the surrogate and the cognate biological reducing systems demonstrated that ADO is selective against Fd and the interaction between Fd and FNR is very important for efficient electron transfer and ADO activity. It has been observed that the interactions between FNR and its protein partner Fd or flavodoxin are essential for efficient assembling and functionality of the formation of FNR-Fd complex [39]. A lot of examples have demonstrated that the interactions between P450s and Fd proteins are significant for P450 activity. For instance, Bell *et al.* tailored an non-physiological Fd to support native-like P450 activity through engineering the surface residues involved in the interaction between Fd and P450 [40]; P450 105D5 from *Streptomyces coelicolor* A3(2) is very selective among *S. coelicolor* Fd proteins, but Fd could interact with the surrogate FNRs from *P. putida* and spinach [22]; With *S. griseolus* P450 105A1 and 105B1, either of two Fd proteins could reconstitute P450 activity, but each Fd worked at least somewhat faster with its cognate P450 [41].

#### Using *n*-heptanal as the substrate

*k*_*cat*_ values of ADO 1593 against *n*-heptanal were determined using the cognate reducing system and the chemical one respectively under saturating concentration of *n*-heptanal (2 mM, Estimated *K*_*m*_ value of ADO1593 for *n*-heptanal was 224 ± 40 μM employing the chemical reductants.). ADO 1593 had *k*_*cat*_ value of 0.44 ± 0.02 min^-1^ in the presence of the homologus Fd/FNR system, whereas *k*_*cat*_ value was 0.12 ± 0.02 min^-1^ for the NADH/PMS system, close to that of ADO from *Prochlorococcus marinus* (0.17 ± 0.01 min^-1^) [16]. Thus, the turnover number of ADO 1593 using the cognate Fd/FNR electron transfer system is 3.7-fold higher than the chemical one. However, according to the report by Choi *et al*., AurF (*p*-aminobenzoate *N*-oxygenase) showed similar *k*_*cat*_ values when reconstituted with either the chemical reductants (NADH/PMS) or the biological one (Fd/FNR from *Anabaena* sp. PCC7119) [20]. Their results could arise from the fact that the surrogate Fd/FNR reducing system was used, again highlighting the importance of the homologous electron transfer system in supporting greater enzymatic activity.

#### H_2_O_2_ production during *in vitro* ADO-catalyzed reactions

Andre *et al.* suggest that the inhibitory H_2_O_2_ formation is due to uncoupled electron transfer from NADPH to O_2_[18]. We directly compared H_2_O_2_ concentration for the cognate electron transfer system and the heterologous one using *n*-hexadecanal as the substrate in order to see whether there is any difference in H_2_O_2_ production between these two biological reducing systems. Interestingly, we observed that the cognate reducing system produced less H_2_O_2_ than the heterologous one by 33%, demonstrating that better coupling between FNR:Fd:ADO in the cognate reducing system might lead to more efficient electron transfer and therefore less formation of H_2_O_2_ due to decoupled electron transfer to O_2_. This may be one reason why the cognate electron transfer system used yields more product than the heterologous one.

## Conclusions

The cognate electron transfer system including Fd and FNR from cyanobacteria to support ADO activity was identified, cloned, overexpressed, purified, and characterized. For the first time, ADO was functionally *in vitro* reconstituted with the endogenous reducing system from cyanobacteria, which supported higher ADO activity than the surrogate Fd/FNR system and the chemical one, and produced less H_2_O_2_ than the heterologous one. The identified Fd/FNR reducing system offers the platform to study the Fd-ADO recognition and electron transfer in detail, will be potentially useful for further improving ADO activity, and might be applicable to other enzymes requiring the electron transfer system. Our findings here might be significant for further building a more active *in vivo* fatty alk(a/e)ne-biosynthesis system in cyanobacteria and constructing a highly efficient photosynthetic microbial cell factory for production of advanced hydrocarbon biofuels.

## Methods

### Materials

Spinach ferredoxin and ferredoxin-NADP^+^ reductase, horse-heart cytochrome *c*, BSA (Bovine Serum Albumin), NADPH, NADH, *n*-heptanal, potassium ferricyanide, phenazine methosulfate (PMS), ferrous ammonium sulfate, Dess-Martin reagent, *n*-hexadecanol were obtained from Sigma-Aldrich. Oligonucleotides were synthesized by Shanghai Sangon Biotech Co. Ltd (China). The gene encoding ADO (Synpcc7942_1593) from *Synechococcus elongatus* PCC7942 with codon optimization was synthesized and cloned into the vector pBluescript II SK(+) using the restriction sites of *Xho*I (3′-terminal) and *Sma*I (5′-terminal) by Shanghai Sangon Biotech Co. Ltd (China) (Additional file 3), and the *Nde*I restriction site was introduced at 5′-terminal. *Taq* DNA polymerases and all restriction endonucleases were from Fermentas or Takara Biotechnology. The kits used for molecular cloning were from Omega Bio-tek or Takara Biotechnology. Nickel column and the expression vectors were from Novagen. Antibodies and chemical reagents used for Western blot were from Tiangen (China). Amicon YM10 membrane was from Millipore. The Amplex Red Hydrogen Peroxide/Peroxidase Assay Kit was purchased from Invitrogen.

### Bacterial strains, plasmids, and media

*E. coli* DH5α was used for routine DNA transformation and plasmid isolation. *E. coli* BL21(DE3) was utilized for protein overexpression. *E. coli* strains were routinely grown in Luria-Bertani broth at 37°C with aeration or on LB supplemented with 1.5% (w/v) agar. 50 μg/ml Kanamycin was added when required. The vector pET-28a(+) was used for subcloning.

### DNA manipulations

General molecular biology techniques were carried out following the standard procedures [42]. Restriction and modification enzymes were used following the recommendations of the manufacturers. DNA fragments were purified from agarose gels using the DNA gel extraction kit. Plasmid DNA was isolated using the plasmid miniprep kit.

The plasmid, where the codon-optimized gene encoding ADO (Synpcc7942_1593) from PCC7942 was inserted into the vector pBluescript II SK(+), was digested with *Nde*I and *Xho*I, and re-cloned into the vectorspET-28a(+) digested with same restriction enzymes.

The gene encoding Fd SynPcc7942_1499 was amplified with the forward primer (5′-GCTCAGCATATGATGGCAACCTACAAGG-3′, the *Nde*I I restriction site underlined) and the reverse primer (5′-GGCTCGCTCGAGTTAGTAGAGGTCTTCTTC-3′, the *Xho*I restriction site underlined) using genomic DNA as a template. The gene encoding FNR SynPcc7942_0978 was amplified with the forward primer (5′-CGCGGCCATATGATGTTGAATGCGAGTGTG-3′, the *Nde*I I restriction site underlined) and the reverse primer (5′-CATTCGCTCGAGGGCTGAACTAGTAGGTTT-3′, the *Xho*I restriction site underlined) using genomic DNA as a template. The PCR products were isolated by agarose electrophoresis and extracted from agarose gel using the DNA gel extraction kit, digested with restriction enzymes *Nde*I and *Xho*I respectively, and re-cloned into the vector pET-28a(+) digested with *Nde*I and *Xho*I, respectively. All constructs were confirmed by DNA sequencing.

### Protein overexpression and purification

The expression constructs pET28a-1593 and pET28a-FNR were transformed into competent *E. coli* BL21(DE3). Protein expression was carried out at 37°C in LB media supplemented with 50 μg/mL kanamycin. The cultures were induced with 1 mM IPTG when OD_600_ reached 0.7, and were shaken at 37°C for additional 3 hours. The his-tagged proteins were purified using Nickel chelating resin according to the manufacturer’s protocol. Apo-ADO was prepared according to the published procedure, and the diferrous form of ADO was reconstituted by the addition of the stoichiometric amounts of ferrous ammonium sulfate to apo-ADO prior to assay [15,16]. Proteins were concentrated with Amicon YM10 membrane (10 kDa cut-off). Protein concentration was determined by the Bradford method using bovine serum albumin as a standard.

The expression construct pET28a-Fd was expressed in *E. coli* BL21(DE3). Protein expression was carried out at 30°C in M9ZB media supplemented with 50 μg/mL kanamycin and 50 μM FeCl_3_[35]. The cultures were induced with 0.2 mM IPTG when OD_600_ reached 0.7, and were shaken at 16°C for 48 hr. His-tagged Fd was purified as above.

SDS-PAGE was performed in 12% polyacrylamide gels, using the low protein molecular weight marker and Coomassie Blue R-250 staining. For Western blot, proteins were transferred from the gel onto the polyvinylidene fluoride (PVDF) membrane using the Mini Trans-Blot Electrophoretic Transfer Cell. The membrane was blocked with 5% (w/v) skimmed milk in TBST (20 mM Tris–HCl, pH 7.5, 150 mM NaCl, 0.05% Tween-20), incubated with the murine monoclonal anti-polyhistidine immunoglobulin G (IgG), rinsed three times with TBST, incubated with the goat anti-mouse IgG conjugated with alkaline phosphatase, rinsed three times with TBST, and detected with the BCIP (5-bromo-4-chloro-3-indolyl phosphate)/NBT (nitro blue tetrazolium) solution.

### Synthesis of *n*-hexadecanal

*n*-Hexadecanal was synthesized following the published procedure [43]. Dess-Martin reagent (0.96 g, 2.3 mmol) in one portion was added to the solution of *n*-hexadecanol (0.5 g, 2.1 mmol) in CH_2_Cl_2_ (40 ml) in an ice bath. After *n*-hexadecanol was completely gone, the reaction was quenched at 0°C by stirring with the saturated NaHCO_3_ solution (40 mL) containing Na_2_S_2_O_3_ (3 g) for 10 min to destroy any unreacted Dess-Martin reagent. The reaction mixture was poured into a separatory funnel and extracted with EtOAc (3 x 40 mL). The organic layers were pooled and washed with brine (50 mL), dried over MgSO_4_ and concentrated. Crude *n*-hexadecanal was purified by silica column chromatography using hexane:ethyl acetate (9:1) as eluent to obtain an oily liquid. The product was confirmed by GC-MS.

### Enzyme assay

All experiments were done at least in duplicate.

(a) For FNR and Fd

Enzymatic assays for FNR and Fd were done on the Beckman Coulter DU 800 UV/Vis Spectrophotometer at 1 ml scale at 25°C.

Ferricyanide reductase activity was measured with NADPH and potassium ferricyanide as the electron donor and acceptor molecules, respectively [27,44]. Assays were performed in 50 mM Tris–HCl (pH 8.0) containing 0.7 mM potassium ferricyanide and different NADPH concentrations (15 – 450 μM) or NADH (2 mM). The reactions were initiated by the addition of 0.02 μM FNR. The absorption decrease at 420 nm (reduction of ferricyanide, ϵ_420_ = 1000 M^-1^ cm^-1^) was recorded to determine steady-state kinetic parameters.

The Fd-mediated cytochrome *c* reductase activity of FNR was measured with Fd and cytochrome *c* acting as the intermediate and terminal electron acceptors [30,41]. Assays were carried out in 50 mM Tris–HCl (pH 7.8) containing 0.01 μM FNR and 50 μM cytochrome *c*. The reactions were started by the addition of NADPH (375 μM). Steady-state kinetic parameters for the Fd-dependent cytochrome *c* reductase activity were determined by varying the concentrations of Fd (3 – 80 μM) and monitoring the resulting absorption increases at 550 nm (reduction of cytochrome *c*, ϵ_550_ = 19,100 M^-1^ cm^-1^).

(b) For ADO 1593

#### n-Hexadecanal used as the substrate

The typical reaction contained 20 μM ADO 1593, 80 μM ferrous ammonium sulfate, 150 μM *n*-hexadecanal in 500 μL of 100 mM HEPES (pH 7.2) containing 0.1 M KCl and 10% glycerol. *n*-Hexadecanal was made up as the stock solution in 2% Triton X-100 containing 150 μM BSA [16]. The biological reducing system is comprised of either 10.26 μM Fd from spinach or PCC7942, 1.8 μM FNR from spinach or PCC7942 and 2 mM NADPH. The chemical reducing system consists of 75 μM phenazine methosulfate (PMS) and 750 μM NADH. For the biological reducing system, four groups of experiments were set up: (a) Fd_spinach_ and FNR_spinach_; (b) Fd_PCC7942_ and FNR_PCC7942_; (c) Fd_spinach_ and FNR_PCC7942_; (d) Fd_PCC7942_ and FNR_spinach_. The reactions were incubated at room temperature for 1 hr at 200 rpm, quenched by the addition of 500 μL ethyl acetate and vortexed to extract the hydrocarbon product and the unreacted substrate. One μL of the ethyl acetate layer was injected into GC-MS for analysis. Enzymatic conversion of *n*-hexadecanal into *n*-pentadecane was quantified using the calibration plot of *n*-pentadecane.

An Agilent 5975C GC-MS system equipped with a quadrupole mass detector was used to detect and quantify the hydrocarbons formed in the enzymatic reactions. The column employed for hydrocarbon analysis was a HP-INNOWax capillary column (30 m × 0.25 mm × 0.25 μm). The ethyl acetate extracts of the reaction mixtures were used for GC-MS analysis. The flow rate of the helium carrier gas was 1.0 mL/min and the inlet temperature was maintained at 250°C. Injections were made in the splitless mode and a total flow of 50 mL/min. The interface temperature was maintained at 250°C. The oven temperature was held at 40°C for 5 min and then increased to 200°C at 25°C/min and finally maintained at 240°C for 15 min. Chromatographic data were analyzed using the associated software.

#### n-heptanal used the substrate

For determining *k*_*cat*_ value of ADO 1593 against *n*-heptanal, assays were performed in 1.5 mL gastight vials with a total volume of 500 *μ*L. The reactions contained 2 mM *n*-heptanal in 100 mM HEPES buffer (pH 7.2) containing 100 mM KCl, 10% glycerol and 4% DMSO, 5 *μ*M ADO, 20 *μ*M ferrous ammonium sulphate, 10.26 μM Fd and 1.8 μM FNR from PCC7942, 2 mM NADPH (for the cognate reducing system), or 15 *μ*M ADO, 60 *μ*M ferrous ammonium sulphate, 75 *μ*M PMS, 750 μM NADH (for the chemical reducing system). The *n*-heptanal stock solution was freshly made in DMSO. After the addition of all the components, the reactions were shaken at 220 rpm at 37°C. To determine the amount of *n*-hexane produced, a sample of the headspace was collected using a gastight sample lock Hamilton syringe and analysed by GC. The amount of *n*-hexane produced was quantified by the standard curve of known concentrations of *n*-hexane.

GC analysis was performed using an Agilent 7890A instrument equipped with a flame ionization detector and a HP-INNOWax capillary column (30 m × 0.32 mm × 0.25 μm). The flow rate of the nitrogen carrier gas was 1.1 mL/min and the inlet temperature was maintained at 250°C. Injections were made in the split mode with a split ratio of 2:1 and a total flow of 2 mL/min. The oven temperature was held at 80°C for 2 min and then increased to 180°C at 20°C/min, followed by increasing to 210°C at 30°C/min, and finally maintained at 210°C for 2 min. The FID detector was at 300°C with a continuous flow of H_2_ at 30 mL/min and air at 350 mL/min. Chromatographic data were analyzed using the HP Chem station software.

#### Determination of H_2_O_2_ production during ADO-catalyzed reactions using n-hexadecanal used as the substrate

ADO-catalyzed reactions were set up as above for the cognate reducing system and the surrogate one. After 15 min, H_2_O_2_ production was determined on Synergy HT Multi-Mode Microplate Reader with the Amplex Red Hydrogen Peroxide/Peroxidase Assay Kit according to the manufacturer’s protocol.

## Abbreviations

ACP: Acyl carrier protein; ADO: Aldehyde-deformylating oxygenase; Fd: Ferredoxin; FNR: Ferredoxin-NADP^+^ reductase; MeOPMS: 1-methyoxy-5-methylphenazinium methylsulfate; P450: Cytochrome P450; PMS: Phenazine methosulfate.

## Competing interests

The authors declare that they have no competing interests.

## Authors’ contributions

JZ, XL, and JJL designed the experiments. JZ performed the experiments, including gene cloning, overexpression, purification, characterization, and enzymatic assays. JZ, XL, and JJL drafted the manuscript. All authors read and approved the final manuscript.

## Supplementary Material

Additional file 1 **Codon-optimized gene sequence of ADO from *Synechococcus elongatus *PCC7942.** Click here for file

Additional file 2: Figure S1 SDS-PAGE analysis of FNR. **Figure S2**. SDS-PAGE analysis of Fd. Click here for file

Additional file 3: Figure S3A Residues involved in FAD binding in FNR from *Synechocystis sp.* PCC7002 (PDB ID:2B5O). **Figure S3B**. Sequence alignment of FNRs from PCC6803, PCC7002, and PCC7942. Click here for file
