# Supplementary material for: Conversion of fatty aldehydes into alk (a/e)nes by in vitro reconstituted cyanobacterial aldehyde-deformylating oxygenase with the cognate electron transfer system
Source: Biotechnol Biofuels. 2013 Jun 8;6:86. doi: 10.1186/1754-6834-6-86 (PMC3691600; doi:10.1186/1754-6834-6-86)
Supplement: Additional file 1 — Codon-optimized gene sequence of ADO from Synechococcus elongatus PCC7942. [file 1754-6834-6-86-S1.pdf]

Additional file 2

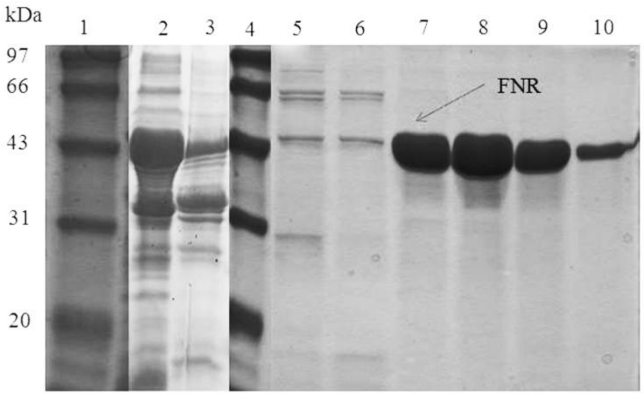

Figure 1 SDS-PAGE analysis of FNR. Lane 1 and 4, low protein molecular weight marker; Lane 2, crude supernatant for over-expressed FNR; Lane 3, cell debris; Lane 5, 6, eluents of buffer A + 80 mM imidazole; Lane 7 - 10, eluents of buffer A + 120 mM imidazole.

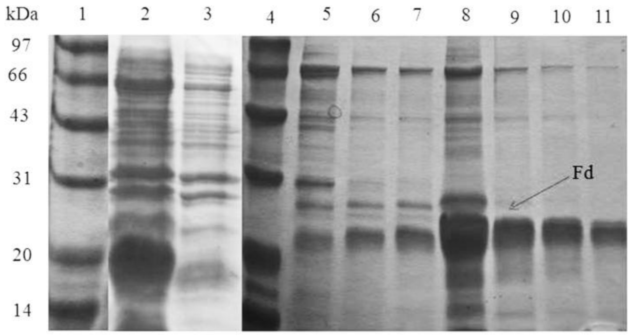

Figure 2 SDS-PAGE analysis of Fd. Lane 1 and 4, low protein molecular weight marker; Lane 2, crude supernatant for over-expressed Fd; Lane 3, cell debris; Lane 5 and 6, eluents of buffer A + 80 mM imidazole; Lanes 7 - 11, eluents of buffer A + 120 mM imidazole.
