# Supplementary material for: Conversion of fatty aldehydes into alk (a/e)nes by in vitro reconstituted cyanobacterial aldehyde-deformylating oxygenase with the cognate electron transfer system
Source: Biotechnol Biofuels. 2013 Jun 8;6:86. doi: 10.1186/1754-6834-6-86 (PMC3691600; doi:10.1186/1754-6834-6-86)
Supplement: Additional file 2: Figure S1 — SDS-PAGE analysis of FNR. Figure S2. SDS-PAGE analysis of Fd. [file 1754-6834-6-86-S2.pdf]

Additional file 3

A

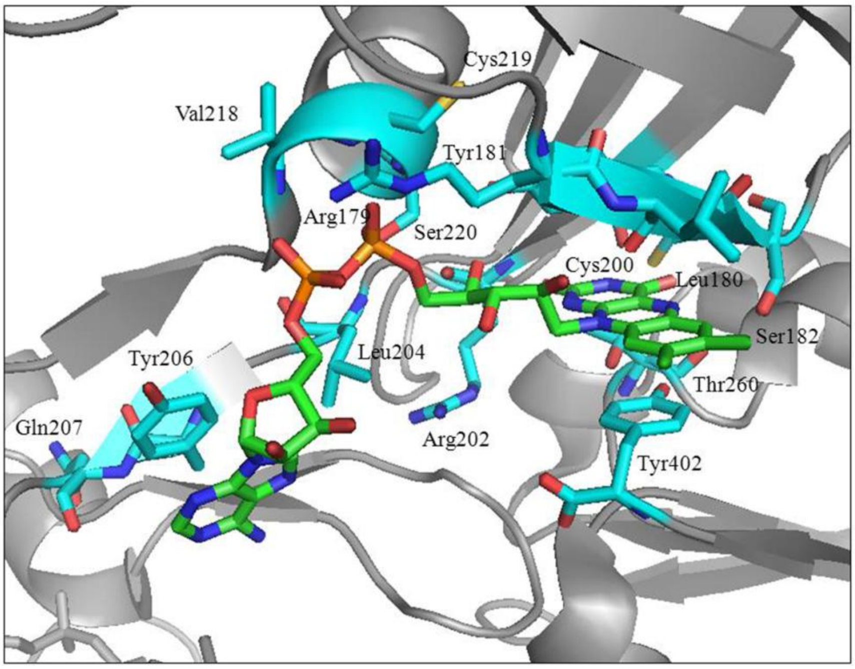

Figure 3A Residues involved in FAD binding in FNR from *Synechocystis sp.* PCC7002 (PDB ID：2B5O).

B

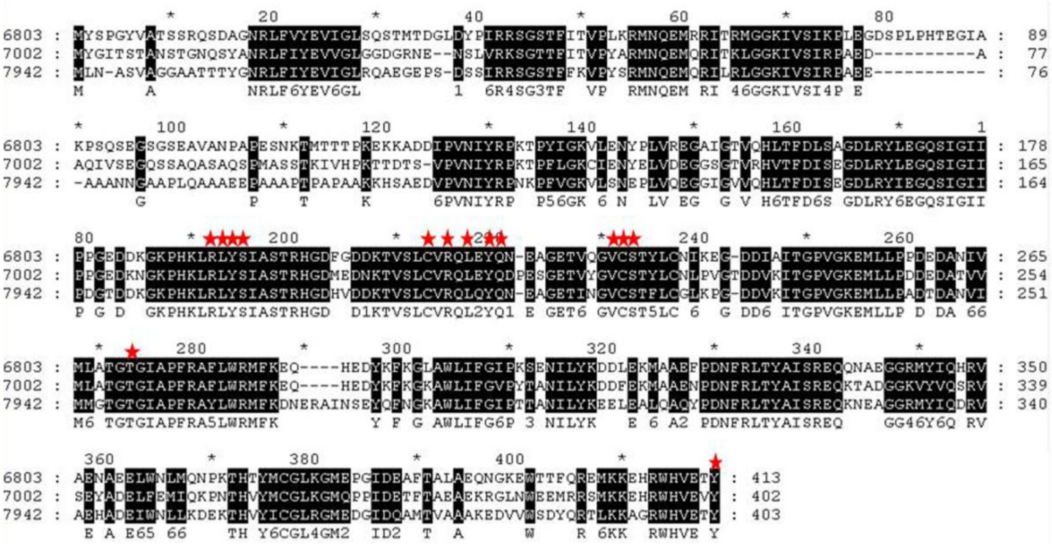

Figure 3B Sequence alignment of FNRs from PCC6803, PCC7002, and PCC7942. Residues involved in FAD binding are completely conserved, and indicated with red stars.
