# Supplementary material for: Conversion of fatty aldehydes into alk (a/e)nes by in vitro reconstituted cyanobacterial aldehyde-deformylating oxygenase with the cognate electron transfer system
Source: Biotechnol Biofuels. 2013 Jun 8;6:86. doi: 10.1186/1754-6834-6-86 (PMC3691600; doi:10.1186/1754-6834-6-86)
Supplement: Additional file 3: Figure S3A — Residues involved in FAD binding in FNR from Synechocystis sp. PCC7002 (PDB ID:2B5O). Figure S3B. Sequence alignment of FNRs from PCC6803, PCC7002, and PCC7942. [file 1754-6834-6-86-S3.pdf]

Additional file 1

Codon-optimized gene sequence of ADO from *Synechococcus elongates* PCC7942

ATGCCGCAGCTGGAGGCGTCTCTGGAACTGGATTTTCAGAGCGAGTCCTAT

AAAGACGCGTACTCCCGCATCAACGCCATCGTGATTGAAGGTGAGCAGGA

AGCATTTGATAACTATAACCGTCTGGCAGAAATGCTGCCGGATCAACGCGA

CGAACTGCATAAACTGGCGAAAATGGAACAGCGCCACATGAAAGGCTTCA

TGGCTTGCGGCAAGAATCTGAGCGTTACGCCAGATATGGGTTTCGCGCAAA

AATTCTTCGAACGTCTGCACGAAAACTTTAAAGCCGCTGCTGCAGAAGGTA

AAGTTGTGACCTGTCTGCTGATCCAGTCTCTGATTATCGAGTGCTTCGCTAT

CGCTGCATACAACATCTACATCCCGGTCGCTGATGCGTTCGCACGTAAAATC

ACCGAAGGTGTGGTTCGTGACGAATACCTGCACCGTAACTTCGGCGAAGA

ATGGCTGAAAGCGAATTTCGATGCTTCTAAGGCAGAGCTGGAAGAGGCGA

ACCGTCAGAACCTGCCTCTGGTATGGCTGATGCTGAACGAAGTTGCAGACG

ACGCTCGCGAACTGGGCATGGAACGTGAATCTCTGGTTGAAGACTTCATGA

TTGCCTACGGCGAAGCCCTGGAAAACATCGGTTTTACCACTCGTGAGATTA

TGCGTATGTCCGCCTATGGTCTGGCGGCGGTATAA
